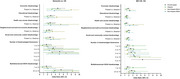# Associations of social determinants of health (SDOH) with dementia and mild cognitive impairment (MCI), the HABS‐HD Study

**DOI:** 10.1002/alz70860_096396

**Published:** 2025-12-23

**Authors:** Shanshan Wang, Uyen‐sa Nguyen, Zhengyang Zhou, Sid E. O'Bryant, Kristine Yaffe, Leigh A. Johnson, Rajesh Nandy, The Health and Aging Brain Study (HABS‐HD) Study Team

**Affiliations:** ^1^ University of North Texas Health Science Center, Fort Worth, TX, USA; ^2^ Institute for Translational Research, University of North Texas Health Science Center, Fort Worth, TX, USA; ^3^ Department of Psychiatry, University of California San Francisco, San Francisco, CA, USA; ^4^ Department of Neurology, University of California, San Francisco, San Francisco, CA, USA; ^5^ University of California San Francisco / San Francisco VA Medical Center, San Francisco, CA, USA

## Abstract

**Background:**

The present study aims 1) to examine the associations of social determinants of health (SDOH) with mild cognitive impairment (MCI) and dementia; and 2) to assess whether the associations differentiate among Hispanics, non‐Hispanic Blacks, and non‐Hispanic Whites.

**Methods:**

Data from the Health & Aging Brain Study‐Health Disparities (HABS‐HD) study were used. A total of 3229 participants were included in the final analyses. A total of 16 indicators were recognized as SDOH: 1) annual household income, 2) occupation, 3) retirement status, 4) education attainment, 5) health insurance, 6) primary care physicians, 7) residence/housing, 8) Area Deprivation Index (ADI), 9) marital status, 10) Interpersonal Support Evaluation List (ISEL) shortened version score, 11) chronic stress burden, 12) time living in the US, 13) potential nativity, 14) primary language, 15) bilingualism, and 16) Short Acculturation Scale for Hispanics (SASH) language use subscale score. Five dimensional SDOH disadvantages were defined based on 16 identified indicators: 1) economic disadvantage, 2) education disadvantage, 3) health care access disadvantage, 4) neighborhood disadvantage, and 5) social disadvantage. Multidimensional SDOH disadvantage is defined as having more than one disadvantaged SDOH dimension. The three‐category (dementia, MCI, and cognitively unimpaired) outcome was used. Multinomial logistic regression models were applied, adjusting for age, sex, and APOE4 positivity.

**Results:**

All the five dimensional SDOH disadvantages were significantly associated with higher odds of both dementia and MCI in the overall sample (*p* <0.05) (Figure 1). Non‐Hispanic Black participants had a stronger association between dimensional SDOH disadvantages and dementia. Four out of the five associations (economic disadvantage, educational disadvantage, neighborhood disadvantage, social disadvantage) with MCI were stronger among non‐Hispanic White participants. Dose response associations were observed between the number of dimensional SDOH disadvantages and dementia, as well as the number of SDOH dimensional disadvantages and MCI.

**Conclusion:**

A broad range of SDOH were associated with cognitive impairment, with the strength of these associations varying across different racial/ethnic groups. This suggests the need for tailored strategies to address SDOH issues in racial/ethnic groups.